# Licochalcone B, a Natural Autophagic Agent for Alleviating Oxidative Stress-Induced Cell Death in Neuronal Cells and *Caenorhabditis elegans* Models

**DOI:** 10.3390/ph15091052

**Published:** 2022-08-25

**Authors:** Liqun Qu, Jianhui Wu, Yong Tang, Xiaoyun Yun, Hang Hong Lo, Lu Yu, Wenhua Li, Anguo Wu, Betty Yuen Kwan Law

**Affiliations:** 1 Neher’s Biophysics Laboratory for Innovative Drug Discovery, State Key Laboratory of Quality Research in Chinese Medicine, Macau University of Science and Technology, Macau 999078, China; 2 Sichuan Key Medical Laboratory of New Drug Discovery and Druggability Evaluation, School of Pharmacy, Southwest Medical University, Luzhou 646000, China; 3 Hubei Key Laboratory of Cell Homeostasis, College of Life Sciences, Wuhan University, Wuhan 430072, China

**Keywords:** Licochalcone B, antioxidant, autophagy, apoptosis, reactive oxygen species

## Abstract

Autophagy has been implicated in the regulation of neuroinflammation and neurodegenerative disorders. Licochalcone B (LCB), a chalcone from *Glycyrrhiza inflata*, has been reported to have anti-cancer, anti-oxidation and anti-β–amyloid fibrillation effects; however, its effect in autophagy remain un-investigated. In the current study, the potential neuro-protective role of LCB in terms of its anti-oxidative, anti-apoptotic, and autophagic properties upon oxidative stress-induced damage in neuronal cells was investigated. With the production of reactive oxygen species (ROS) as a hallmark of neuroinflammation and neurodegeneration, hydrogen peroxide (H_2_O_2_) was adopted to stimulate ROS-induced cell apoptosis in PC-12 cells. Our findings revealed that LCB reduced cell cytotoxicity and apoptosis of PC-12 cells upon H_2_O_2_-stimulation. Furthermore, LCB increased the level of the apoptosis-associated proteins caspase-3 and cleaved caspase-3 in H_2_O_2_-induced cells. LCB effectively attenuated the level of oxidative stress markers such as MDA, SOD, and ROS in H_2_O_2_-induced cells. Most importantly, LCB was confirmed to possess its anti-apoptotic effects in H_2_O_2_-induced cells through the induction of ATG7-dependent autophagy and the SIRT1/AMPK signaling pathway. As a novel autophagic inducer, LCB increased the level of autophagy-related proteins LC3–II and decreased p62 in both neuronal cells and *Caenorhabditis elegans* (*C. elegans*) models. These results suggested that LCB has potential neuroprotective effects on oxidative damage models via multiple protective pharmacological mechanisms.

## 1. Introduction

A growing body of experimental and clinical evidence has confirmed that the high level of free radicals produced by oxidative stress is one of the pathogenic features in many neuronal disease models. Free radicals produced by oxidative stress affect the structure and function of neuronal cells [1], which leads to the progression of neurodegenerative diseases including Parkinson’s and Alzheimer’s disease [2]. Neurodegenerative diseases are characterized by the atrophy of specific nuclei and progressive loss of function of neurons, but their etiology and pathology remain unclear. Multiple risk factors including aging, external environmental, and internal genetic risk factors contribute cumulatively to the occurrence and progression of neurodegenerative diseases [3]. Generally, apoptosis is considered essential for the normal development and homeostasis of all multicellular organisms in vivo [4]. This form of programmed cell death is also important for the removal of damaged cells, resistance to bacterial infections, or the removal of potentially tumorigenic cells [5]. Therefore, a balanced rate of apoptosis is important for preventing adverse cellular conditions. Upon oxidative stress conditions, autophagy removes damaged organelles and protein aggregates of cells, which is critical for maintaining normal cellular homeostasis [6]. Moreover, failure or impairment of autophagy in cells may cause an accumulation of soluble or aggregated tau protein, a decreased level of Beclin–1, and finally the increased expression of amyloid precursor protein (APP) and β–amyloid, which are related to the pathogenesis of Alzheimer’s disease and oxidative stress conditions [7]. In addition, research has revealed that the preservation of autophagic activity is vital for preventing detrimental intracellular accumulation of damaged molecules [8].

**Figure 1:**
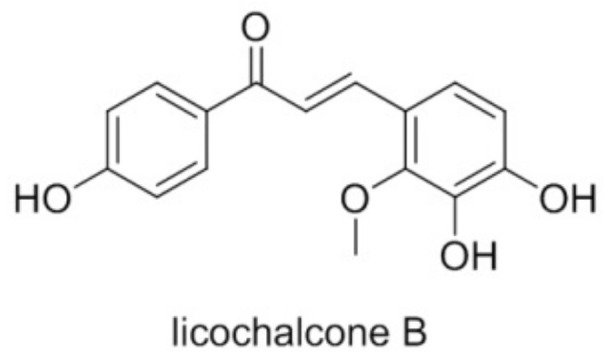
Chemical structure of LCB.

Licochalcone is a major biological active phenolic components of the medicinal herb licorice *Glycyrrhiza inflate* [9]. The anti-oxidative effect of licochalcone A was reported for its suppressive effect on the production of ROS and neuronal apoptosis to exert neuroprotective effects [10]. Licochalcone D protected mouse heart from oxidative damage attributed to the activation of autophagy [11]. Licochalcone E activated antioxidant gene-dependent pathways related to mechanisms of defense against oxidative stress and inflammatory responses [12]. LCB (Figure 1) has been shown to possess anti-oxidative, anti-inflammatory, anti-cancer, cardioprotective, and hepatoprotective activities [13]. The anti-apoptotic effect of LCB was also reported in an alcohol-induced hepatocyte injury model [14]. In fact, many herbal medicines have been reported to exert protective autophagic effects against cellular stressful conditions such as apoptosis and oxidative stress [15]. With the reported pharmacological role of several chemical components of *Glycyrrhiza inflate*, however, the pharmacological role of LCB in autophagy induction remain un-investigated. With the reported protective role of autophagy and apoptosis in neuroprotection [16], this study adopted H_2_O_2_-induced PC-12 cells as an in vitro oxidative stress induction system for the investigation of the regulation of both autophagy and apoptosis by LCB. PC-12 cells, originated from pheochromocytoma and isolated from rat renal medulla, are neuronal–like and share similar secretary properties and embryological origins with neurons [17]. They can be induced by nerve growth factor (NGF) to differentiate into neuronal–like cells; therefore, it is widely adopted as the neuronal model for the study of neuropathic pain, neurotoxicity, and neuro–degenerative diseases [18]. In order to provide a theoretical basis for the development of a novel natural autophagic agent for its possible protective effect in neuroprotection, the pharmacological effects of LCB in term of its anti-apoptotic and autophagic ability were studied in both PC-12 cells and *C. elegans* models.

## 2. Results

### 2.1. LCB Attenuated H_2_O_2_-Induced Cell Death in PC-12 Cells

**Figure 2:**
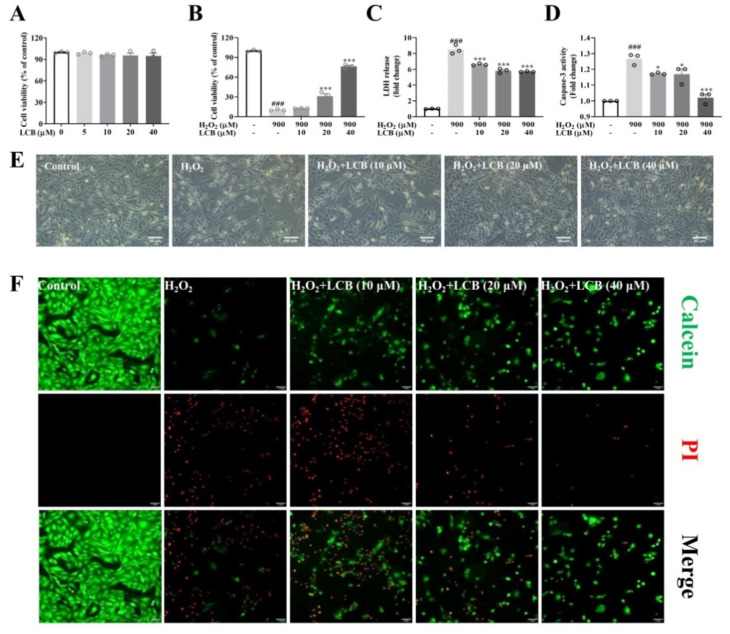
LCB attenuated H_2_O_2_-induced cellular oxidative damage in PC-12 cells. (**A**) The cell viability of PC-12 cells treated with 0 to 40 μM of LCB for 24 h was measured by the MTT assay. (**B**) Cell viability, (**C**) LDH level, and (**D**) Caspase–3 activity were measured in H_2_O_2_ (900 μM)-induced PC-12 cells for 6 h after LCB (10, 20, or 40 μM) pre-treatment for 16 h. ### *p* < 0.001, H_2_O_2_ group vs. control; * *p* < 0.05, *** *p* < 0.001, H_2_O_2_ + LCB group vs. H_2_O_2_ group. (**E**) Cellular morphological changes were evaluated in H_2_O_2_ (900 μM) -induced PC-12 cells for 6 h after pretreatment of LCB for 16 h as indicated. Scale bar = 50 μm. (**F**) Calcein/PI assay was adopted to perform the ICC staining in H_2_O_2_ (900 μM)-induced PC-12 cells for 6 h after pretreatment of LCB for 16 h as indicated. Scale bar = 50 μm.

To investigate the effect of LCB on H_2_O_2_-induced cell death, the establishment of an H_2_O_2_-induced cell death model and the cytotoxicity of LCB were firstly examined in PC-12 cells. Based on the results in Figure 2A and Supplementary Figure S1, while no significant decrease in cell viability was observed in 5 to 40 μM of LCB treatment in PC-12 cells, 900 μM of H_2_O_2_ treatment for 6 h was selected as the experimental conditions of the oxidative stress-induced cell death model in the subsequent experiments. In Figure 2B, the survival rate of PC-12 cells was rescued in a dose-dependent manner after 10 to 40 μM of LCB treatment in the H_2_O_2_-induced cells. With LDH and caspase-3 as the representative cytotoxicity markers and enzymes in the process of apoptosis, significant decreases in the levels of both markers in the H_2_O_2_-induced cells after LCB treatments are shown in Figure 2C and Figure 2D. Furthermore, the protective effect of LCB was evaluated by morphological observation. While obvious cellular shrinkage and reduced numbers of cells were observed in H_2_O_2_-treated PC-12 cells, a higher density of cells with a normal morphological phenotype was observed in the cells pre-treated with LCB before H_2_O_2_ induction (Figure 2E). Furthermore, a calcein/propidium iodide (PI) cytotoxicity assay using two different fluorescent stains was employed to distinguish live cells from dead cells. While green fluorescent calcein AM indicated live cells, propidium iodide (PI) stained dead cells in red. As shown in Figure 2F, although the number of live cells was severely decreased in the H_2_O_2_-induced group when compared to the control group, the number of dead cells was decreased after LCB treatments. These results confirmed that LCB attenuated H_2_O_2_-induced cell death in PC-12 cells.

### 2.2. LCB Inhibited H_2_O_2_-Induced Oxidative Stress

**Figure 3:**
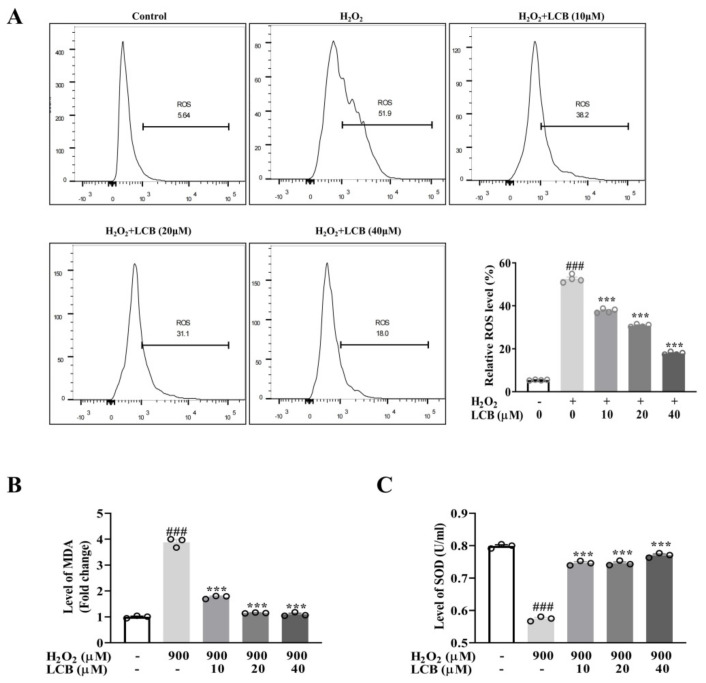
Effect of LCB on H_2_O_2_-induced oxidative stress in PC-12 cells. (**A**) PC-12 cells were pretreated with 25 μM of LCB for 16 h before exposure to 900 μM H_2_O_2_ for 6 h. The mean fluorescence intensity of intracellular ROS was quantified using flow cytometry. ### *p* < 0.001, H_2_O_2_ group vs. control; *** *p* < 0.001, H_2_O_2_ + LCB group vs. H_2_O_2_ group. (**B**,**C**) PC-12 cells were pretreated with 10, 20, or 40 μM of LCB for 16 h before exposure to 900 μM H_2_O_2_ for 6 h. MDA and SOD levels were conducted in H_2_O_2_-induced PC-12 cells (with or without the treatment of LCB) as the indicated conditions.

Reactive oxygen species (ROS) is one of the crucial detrimental factors leading to neuronal cell damage and neurodegenerative diseases. Upon induction by H_2_O_2_, ROS production can be detected by DCFH–DA, which is oxidized by intracellular ROS to generate fluorescent DCF that can be detected by fluorescence microscopy and flow cytometry. PC-12 cells pre-incubated with increasing concentrations of LCB followed by the addition of H_2_O_2_ were performed. As shown by fluorescence microscopic analysis in Supplementary Figure S2, the results showed that while H_2_O_2_ increased the fluorescence intensity of DCF, LCB diminished the fluorescence intensity of DCF in a dose-dependent manner. The increased production of ROS and the anti-oxidative effect of LCB in H_2_O_2_-induced PC-12 cells were further confirmed by quantitating DCF fluorescence intensity using flow cytometry (Figure 3A). Furthermore, important markers of oxidative stress induced by ROS production including malondialdehyde (MDA) and superoxide dismutase (SOD) were measured. As seen in Figure 3B, the MDA level was increased 3.8–fold after H_2_O_2_ challenge, while LCB (10 to 40 μM) attenuated the level of MDA to a level comparable to the control group. Consistently, SOD levels were significantly increased from 0.57 U/mL (H_2_O_2_ group) to around 0.77 U/mL (H_2_O_2_ + LCB 40 μM group) after LCB treatment (Figure 3C). Thus, LCB was shown to suppress the level of ROS generation and lipid peroxidation and recover the level of superoxide dismutase for its protective anti-oxidation effect. 

### 2.3. LCB Protected PC-12 Cells from H_2_O_2_-Induced Apoptotic Cell Death

**Figure 4:**
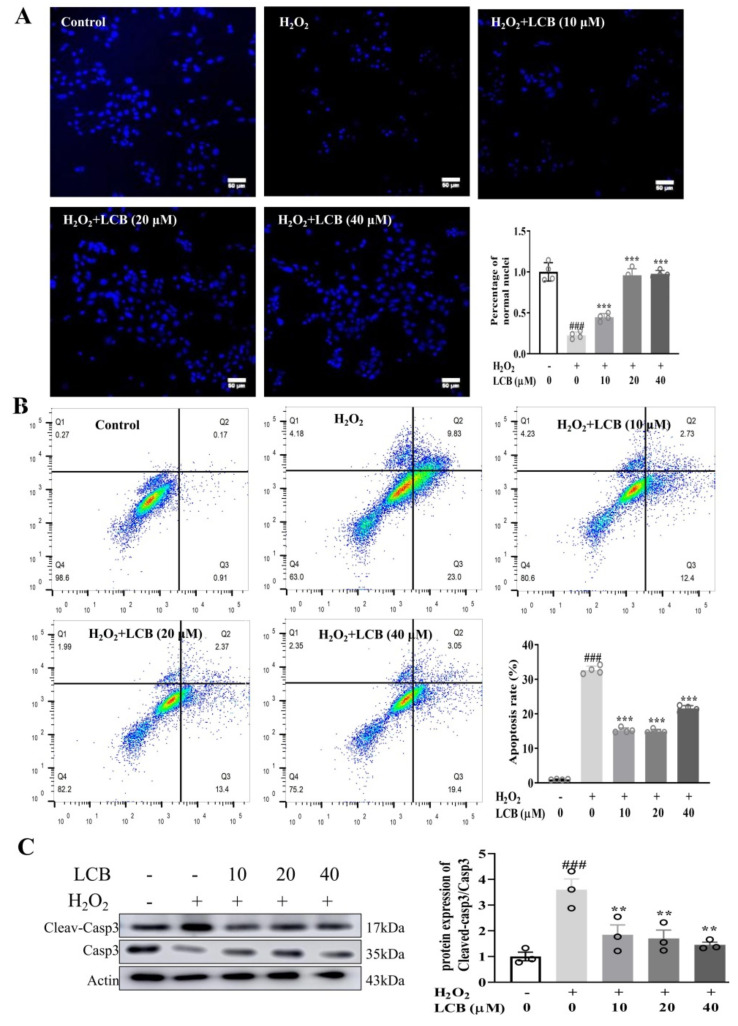
LCB protected PC-12 cells from apoptosis induced by H_2_O_2_. (**A**) PC-12 cells were pretreated with 10, 20, or 40 μM of LCB for 16 h before exposure to 900 μM H_2_O_2_ for 6 h. Hoechst 33342 staining was performed in cells for 10 min. Scale bar = 50 mm. *** *p* < 0.001 (**B**) PC-12 cells were pretreated with 10, 20, and 40 μM of LCB for 16 h before exposure to 900 μM H_2_O_2_ for 6 h. Apoptotic cells detected by using annexin/propidium iodide (PI) double staining were quantitated by flow cytometry. (**C**) PC-12 cells were pretreated with LCB (10 to 40 μM) for 16 h before being subjected to 900 μM H_2_O_2_ for 6 h. Western blot analysis was used to detect the level of cleaved or total caspase-3 protein expression, ** *p* < 0.01, ### *p* < 0.001, H_2_O_2_ group vs. control; *** *p* < 0.001, H_2_O_2_ + LCB group vs. H_2_O_2_ group. The original uncropped images of Western blotting are shown in Supplementary Figure S3.

Recent reports have confirmed that overproduction of ROS can result in the induction of oxidative stress and subsequently apoptosis. Anti–oxidative agents can be applied to inhibit or delay apoptosis and cell death [19]. As revealed by Hoechst 33342 fluorescent staining on DNA and nuclei in Figure 4A, while there was a decrease in the number of stained cells after H_2_O_2_ treatment, the LCB treatment group showed an increase in the number of positive stained cells after H_2_O_2_ induction, suggesting the possible effect of LCB on anti-apoptosis. To further investigate the effect of LCB on H_2_O_2_-induced apoptotic cell death, annexin V/PI was utilized to detect apoptotic cells. As shown in Figure 4B, the percentage of PI–positive (dead) cells was significantly increased after the exposure to H_2_O_2_, while pre-treatment of PC-12 cells with 10 to 40 μM of LCB before H_2_O_2_ exposure showed a significant reduction in the percentage of PI–positive cells. Consistently, while the expression of cleaved apoptotic protein caspase-3 exhibited a significant increase in H_2_O_2_-induced PC-12 cells, the presence of LCB decreased the level of cleaved caspase-3 (Figure 4C). These results indicated that LCB protects PC-12 cells from H_2_O_2_-induced damage through the anti-apoptotic signaling pathway.

### 2.4. LCB Induced Autophagy in PC-12 and C. elegans Models

**Figure 5:**
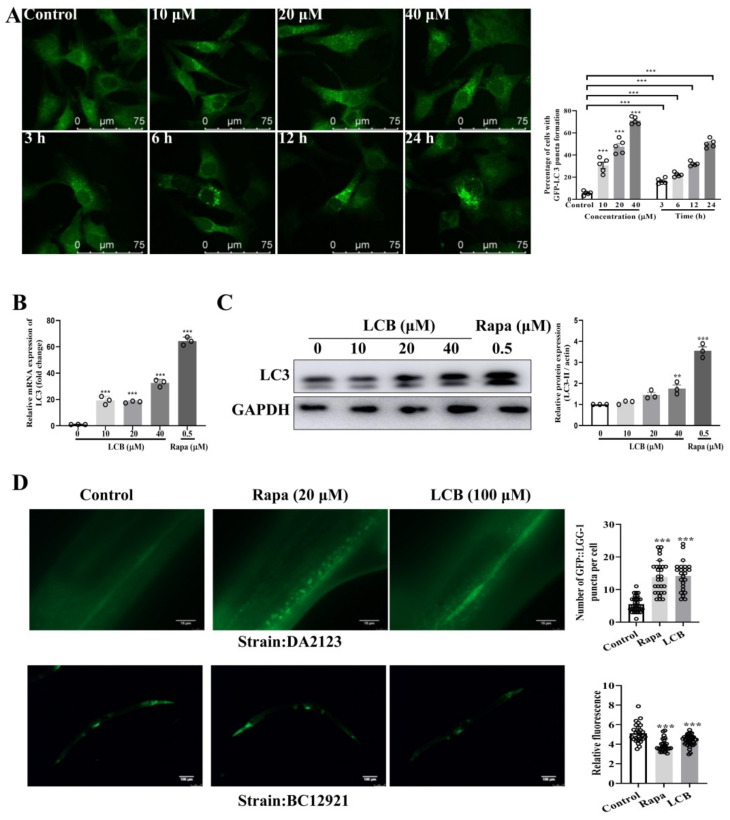
LCB enhanced autophagy in GFP–LC3–U87 stable cells and *C. elegans* models. (**A**) GFP–LC3–U87 cells were exposed to various concentrations of LCB (10 to 40 μM) for 24 h or various treatment durations (3 to 24 h) of LCB (20 μM), as indicated. Punctate GFP–LC3 were calculated from the counts in five randomly selected visual fields. Representative fluorescence images with punctate GFP–LC3 are shown. Scale bar = 75 μm, *** *p* < 0.001. (**B**) Real time (RT)–PCR and (**C**) Western blot for the expression of LC3–II/I after treatment of LCB or rapamycin for 24 h. ** *p* < 0.01, *** *p* < 0.001. (**D**) Two autophagic model of *C. elegans*: LGG–1::GFP and SQST–1/p62::GFP. The former model shows the number of fluorescent spots (LGG–1) indicating autophagic activity (positive correlation). The latter model represents fluorescence intensity of the autophagic substrate (p62), which is negatively correlated to the autophagic activity. Rapa (rapamycin) (20 uM) was used as the positive control. *** *p* < 0.001. The original uncropped images of Western blotting are shown in Supplementary Figure S4.

As a key catabolic process for the lysosomal degradation of dysfunctional cytoplasmic components, autophagy is activated in response to oxidative stress for the protection of cells from apoptosis. In this study, the GFP–microtubule-associated protein light chain 3 (LC3) U87 stable cells were adopted to evaluate the autophagic effect of different concentrations (10–40 μM) and treatment times (0–24 h) of LCB. With the level of the autophagosomal LC3–II as the key marker for autophagic activity, LC3–II levels were detected by using both immunoblotting and immunofluorescence methods after LCB treatments. As shown in Figure 5A, the percentage of cells with GFP–LC3–II puncta formation was increased significantly in both time– and dose-dependent manners after LCB treatments. Figure 5B and Figure 5C confirmed that LCB significantly increased the expression of LC3–II autophagic markers in PC-12 cells. To justify the novel autophagy role of LCB in the study, *C. elegans* was adopted as the in vivo model organism. Due to its small size and transparency for microscopy, together with the rapid growth and high fertility nature, *C. elegans* has emerged in the past decade as a well–adopted model to study various physiological and pathological contexts such as autophagy, apoptotic cell death, and RNA interference studies. Importantly, with its characteristic of simplicity, *C. elegans* was adopted for the study of autophagy, especially on tissue specificity and developmental processes [20]. To begin, two autophagic models of *C. elegans* expressing either GFP–LGG1/LC3 (LGG–1/LC3::GFP) or GFP–p62 (SQST–1/p62::GFP) were adopted. Consistent with the increased level of LC3–II in PC-12 cells and *C. elegans* (Figure 5D), a decreased level of the autophagic substrate p62 was observed in the *C. elegans* model (Figure 5D) after LCB treatments, confirming the induction of autophagic flux in both in vitro and in vivo models. 

### 2.5. The Expression Profile of Autophagy–Related Genes in SH–SY5Y Human Cells after Treatment of LCB 

**Figure 6:**
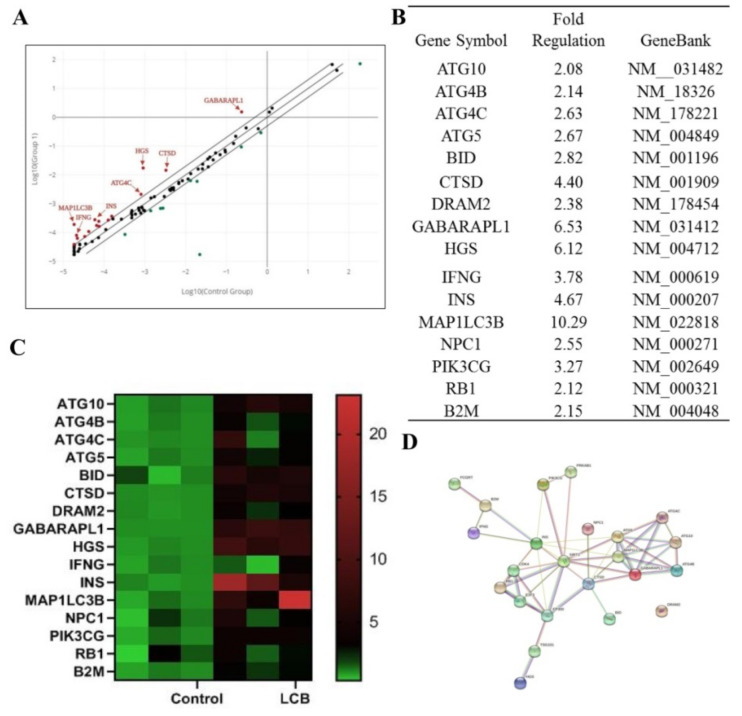
RT–PCR array on the gene expression level of autophagy-related genes in SH–SY5Y cells with or without 20 uM LCB-treatment for 24 h. (**A**) Scatter plot and (**B**) table showing the gene expression level of SH–SY5Y cells under control or LCB treatments. (**C**) Heap map and (**D**) STRING database predicted differential autophagic genes and its possible pathway on AMPK/SIRT1 activation upon LCB treatment.

To explore the signaling pathway of autophagy activated by LCB, SH–SY5Y cells were treated with LCB for 24 h. RNA extracted from SH–SY5Y cells with or without LCB-treatment was converted to cDNA for RT–PCR array analysis on 84 selected autophagy-related genes with the expression profile shown in Figure 6A and Figure 6B. As indicated in the LCB-treated group, 16 autophagic genes including ATG10, ATG4B, ATG4C, ATG5, BID, CTSD, DRAM2, GABARAPL1, HGS, IFNG, INS, MAP1LC3B, NPC1, PIK3CG, RB1, and B2M were found upregulated more than two fold when compared to the control group. As shown in Figure 6C, a heat map was adopted for differential genes expression showing that the mRNA level of the above 16 genes increased after LCB treatments. To further explore the mechanism of LCB–activated autophagy, the String database was adopted to search for its possible associated proteins and to predict that AMPK/SIRT1 signaling pathways may be linked to these 16 upregulated genes (Figure 6D).

### 2.6. LCB Activated Autophagy via the AMPK/SIRT1 Signaling Pathway in PC-12 Cells

**Figure 7:**
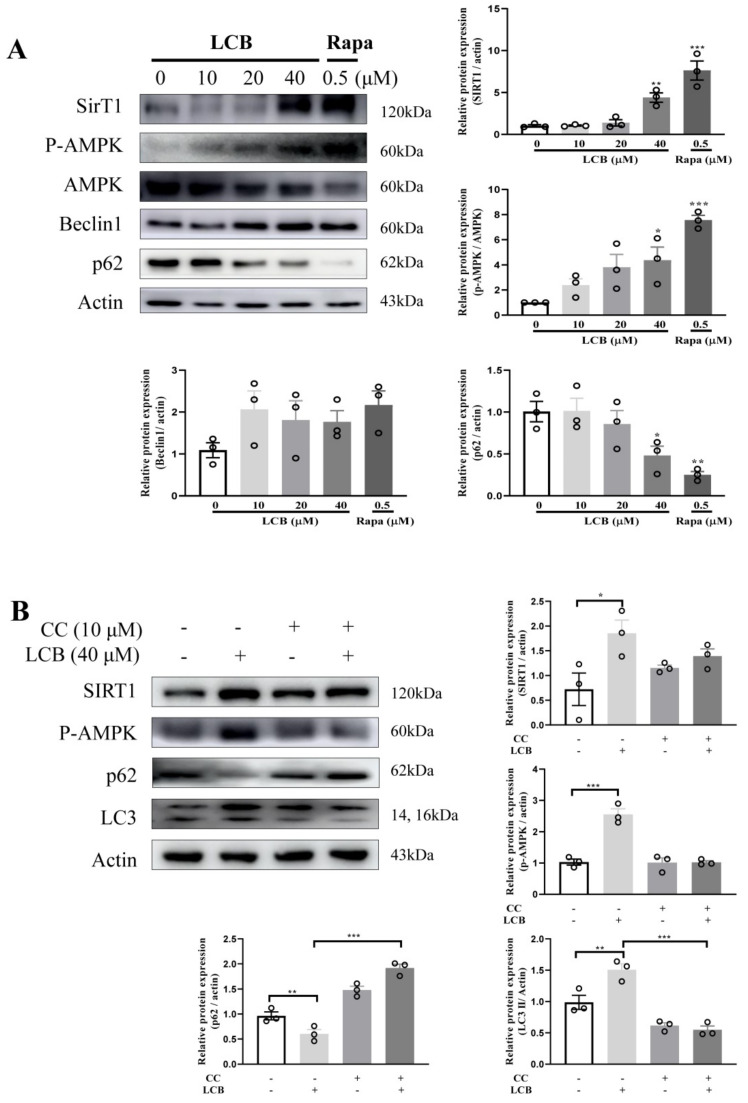
LCB activates autophagy via the SIRT1/AMPK pathway. (**A**) PC-12 cells were treated with LCB under the indicated concentrations for 24 h. After treatment, cell lysates were harvested for the analysis of SIRT1, P–AMPK, Beclin–1, and p62 by Western blot. (**B**) PC-12 cells were treated with LCB or cotreated with LCB and CC under the indicated concentrations for 24 h. After treatment, cell lysates were harvested for the analysis of SIRT1, P–AMPK, and p62 and LC3 by Western blot. Bar chart indicating the normalization of the interest proteins to β–actin. * *p* < 0.05, ** *p* < 0.01, *** *p* < 0.001. The original uncropped images of Western blotting are shown in Supplementary Figure S5.

The cellular energy sensor, AMPK, and SIRT1, which plays an important role in activating autophagy were investigated. As shown in Figure 7A, the phosphorylation of AMPK at Thr272 and the SIRT1 protein level were increased by LCB in a dose-dependent manner. While Beclin–1 is one of the central regulators for the initiation stage of autophagosome membrane formation, p62 is one of the selective substrates for autophagy in the formation of cytoplasmic inclusion. By using rapamycin (rapa) as the positive control, Figure 7A shows that LCB was able to upregulate Beclin–1 and downregulate p62. Compound C (CC), a specific inhibitor of AMPK, abolished the effect of LCB on the protein expression of phosphorylation of AMPK and SIRT1 (Figure 7B). Consistently, the autophagy markers including LC3II and p62 (Figure 7B), which were modulated by LCB, were downregulated and upregulated, respectively, after CC treatments. Taken together, LCB may induce autophagy partially via the AMPK/SIRT1 signaling pathway in PC-12 cells.

### 2.7. LCB Reduced H_2_O_2_-Induced Apoptosis through AMPK/SIRT1–Mediated Autophagy

**Figure 8:**
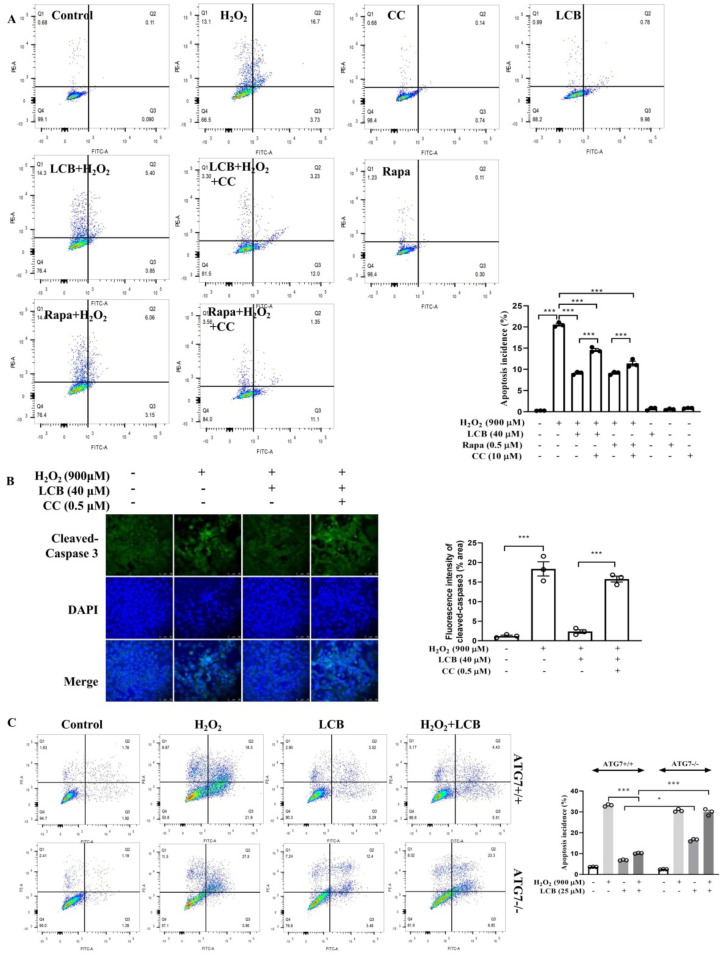
LCB rescued H_2_O_2_-induced apoptosis via enhancing autophagy. (**A**) PC-12 cells were pretreated with CC for 1 h and then treated with LCB or Rap for 16 h in the presence or absence of CC before the addition of H_2_O_2_ for 2 h. Cells were then collected and analyzed by flow cytometry using the Annexin VFITC/PI Apoptosis Detection Kit. *** *p* < 0.001. (**B**) PC-12 cells were pretreated with CC for 1 h and then treated with LCB or Rap for 16 h in the presence or absence of CC before adding H_2_O_2_ for 2 h. Cells were then fixed with 4% PFA. The representative images with GFP–cleaved–caspase-3 were captured. Scale bar: 50 μm. *** *p* < 0.001. (**C**) Representative flow cytometric images showing the percentage of apoptotic cells in both H_2_O_2_-induced ATG7−/− and ATG7+/+ cells with or without the presence of 25 μM of LCB were presented; * *p* < 0.05, *** *p* < 0.001.

To further confirm the protective role of LCB-induced autophagy in H_2_O_2_-induced apoptotic cell death, the autophagic incidence of cells after LCB treatment was measured in an H_2_O_2_-induced cell model. As shown in Supplementary Figure S6, the MDC staining results demonstrated that autolysosomes were accumulated dose-dependently in the H_2_O_2_-induced PC-12 cells after LCB treatments. Therefore, the mechanistic pathway of LCB-induced autophagy and its effect on the apoptosis rate of H_2_O_2_-induced PC-12 cells were further investigated. While LCB significantly suppressed H_2_O_2_-induced apoptosis of PC-12 cells, CC abolished the anti-apoptotic effects of LCB as revealed by annexin V–PI staining flow analysis and the immunofluorescence assay of cleaved–caspase-3 in H_2_O_2_-induced PC-12 cells (Figure 8A). While the cellular apoptosis of H_2_O_2_-induced PC-12 cells is closely associated with the cleaved–caspase-3 level, LCB was confirmed to suppress protein expression of cleaved–caspase-3 in the presence of H_2_O_2_, as shown in Figure 8B. Moreover, the role of autophagy gene ATG7 in LCB-induced autophagy was investigated in ATG7 wild–type and ATG7-deficient mouse embryonic fibroblasts (MEFs). As shown by the flow cytometry result in Figure 8C, LCB lowered significantly the apoptosis rate of H_2_O_2_-induced wild–type MEFs (ATG7+/+) but not ATG7-deficient MEFs (ATG7−/−). Taken together, these data suggested that LCB rescued cells from H_2_O_2_-induced apoptosis via ATG7 dependent autophagy and the AMPK/SIRT1-related pathway.

## 3. Discussion

**Figure 9:**
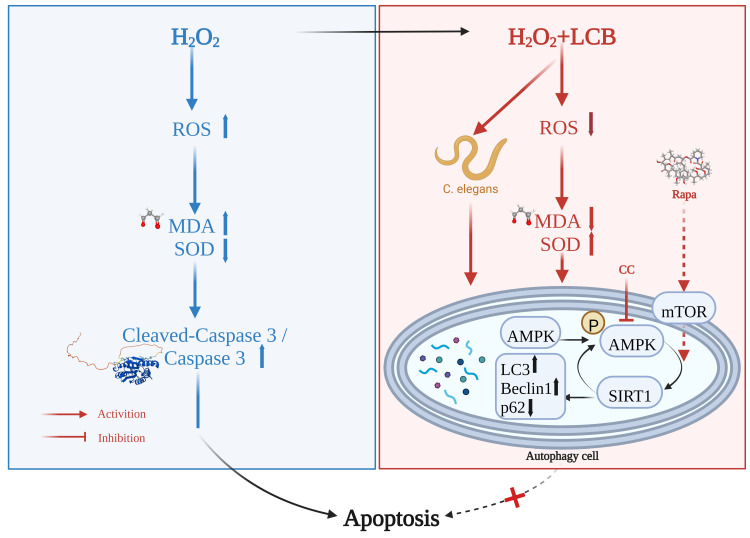
Schematic diagrams depicting the autophagic protective effects of LCB against H_2_O_2_-induced cell apoptosis via the AMPK/SIRT1 autophagy signal pathways. CC, Compound C; Rapa, rapamycin; ROS, reactive oxygen species; MDA, malondialdehyde; SOD, superoxide dismutase.

With the critical role of oxidative stress in the pathogenesis of neuroinflammation, which is highly correlated with neurodegenerative diseases such as Alzheimer’s and Parkinson’s disease, the identification of natural anti-oxidative agents with protective effects in the inhibition of inflammation and apoptotic cell death of the brain is highly desirable [21]. With minimal side effects when compared to synthetic compounds, many natural compounds have been reported for their polypharmacological neuroprotective action via both anti-inflammatory and antioxidant activities. For example, the well–known polyphenol flavonoid, resveratrol, possesses neuroprotective effects via its potent antioxidant and anti-neuroinflammatory activity for the attenuation of neurotoxicity [22]. Licorice root is one of the most widely studied medicinal plants of the world. It is commonly applied as a herbal medicine. It has been traditionally used for the treatment of arthritis, heart diseases, lung diseases, gastric ulcer, and some microbial infections. Among them, a large number of biological active compounds was isolated from the species *Glycyrrhiza* [23]. In this study, our results have reported for the first time that LCB, a chalcone isolated from *Glycyrrhiza inflate*, enhanced autophagy and attenuated apoptotic cell death and oxidative stress, possibly via the modulation of the autophagic markers including SIRT1, AMPK, Beclin–1, LC3, and P62 in either the in vivo PC-12 cells or in vitro *C. elegans* models (Figure 9).

H_2_O_2_ is a reactive oxygen molecule that is involved in the pathogenesis of many neurological diseases and is commonly used as an inducer of oxidative damage and apoptotic cell death in cellular models. Excessive production of free radicals can lead to cellular damage and cause aging and dysfunction of the human body [24]. With the properties of high oxygen demand and metabolic rate, and a relatively low antioxidant defense system, brain tissue is more vulnerable to free radical attack than any other tissues or organs [25]. In the current study, the accumulation of reactive oxygen species in the cellular assay has demonstrated that H_2_O_2_ treatment significantly induced apoptosis in PC-12 cells, and LCB protected PC-12 cells from apoptotic damages, as observed from morphology, apoptosis, and oxidative markers evaluation. Consistently, while the viability of PC-12 cells exposed to 900 μM of H_2_O_2_ were decreased by approximately 50%, LCB pretreatment significantly inhibited damage and increased cell viability upon H_2_O_2_ challenge. With malondialdehyde (MDA) as the final product of lipid peroxidation in cells, the elevation of free radicals can increase the level of MDA; therefore, MDA is commonly adopted as a biomarker of oxidative stress in disease models [26]. In the current H_2_O_2_-induced PC-12 cell model, the increase in both the ROS and MDA level were significantly alleviated after the treatment of LCB, confirming the anti-oxidative role of LCB in cells. With the close correlation of oxidative stress and apoptosis, LCB was confirmed to alleviate H_2_O_2_-induced cell apoptosis, as revealed by decreasing apoptosis-related proteins such as cleaved–caspase-3 in PC-12 cells. Importantly, LCB alleviated apoptosis induced by H_2_O_2_ in ATG7 wild–type MEFs but not in ATG7-deficient MEFs, suggesting that LCB attenuated H_2_O_2_-stimulated cell apoptosis via the autophagy–gene-dependent mechanism. Furthermore, the cytoprotective effects of LCB are related to the regulation of the SIRT1/AMPK signaling pathway.

In fact, autophagy is a widespread biological phenomenon in eukaryotic cells for maintaining normal cellular homeostasis [27] and oxidative status [28]. In neurodegenerative diseases, autophagy removes toxic aggregated proteins and damaged organelles accumulated with ROS. In addition, autophagy also plays a regulatory role in apoptosis, and protects cells from external stresses such as nutrient deprivation or aggregation of pathogenic proteins [29]. Emerging evidence indicates that two programmed cell death modalities, autophagy and apoptosis, can antagonize or promote each other in different scenarios, and they are able to occur sequentially or co–exist in the same cell [30]. While the same inducing factors can induce autophagy or apoptosis in different cells, some molecules involved in autophagy and apoptosis may also intersect and play positive or negative roles in modulating autophagic and apoptotic programmed cell death [31]. 

In the current study, increased formation of autophagy marker (LC3) in both stable GFP–LC3–U87 cells and PC-12 cells were observed. Furthermore, the autophagic effect of LCB was confirmed in two autophagic in vivo model of *C. elegans*: LGG–1::GFP and SQST–1/p62::GFP. Therefore, to further explore the molecular mechanism of LCB-induced autophagy, a PCR array was adopted to assess the expression of 96 autophagy genes (including the house–keeping genes) after LCB treatment. Heap maps showed that the autophagic genes including MAP1LC3B, ATG10, ATG4B, ATG4C, ATG5, BID, CTSD, DRAM2, GABARAPL1, HGS, IFNG, INS, NPC1, PIK3CG, RB1, and B2M were found upregulated more than two fold when compared to the control group. Interesting, these autophagy genes are closely associated with SIRT1 and AMPK signaling pathways. SIRT1 plays a major role in the regulation of several transcription factors related to autophagy, and AMPK activates autophagy by acting as the cellular energy sensor [32]. Previous studies reported that the natural compound, resveratrol, activated AMPK–SIRT1 autophagy for the modulation of Parkinson’s disease in a cell model [33]. Quercetin inhibited oxidative stress responses of high fat diet-induced atherosclerosis of rat by AMPK/SIRT1/NF–κB signaling [34]. With AMPK/SIRT1 working as the important signaling sensor of oxidative stress, AMPK-dependent GAPDH phosphorylation triggered Sirt1 activation and is required for autophagy induction upon glucose starvation [35]. In this study, LCB was confirmed to increase p–AMPK at Thr272 and SIRT1 protein expression. With the fact that Beclin–1 is also one of the central regulators for the initiation stage of autophagosome membrane formation, p62 can act as a receptor for vesicles that are going to be degraded by autophagy [36]. LCB increased protein expression of Beclin–1 but decreased p62. Consistently, CC could abolish the effect of LCB on the protein expression of phosphorylation of AMPK, SIRT1, LC3II, and p62, suggesting that LCB may induce autophagy via the AMPK/SIRT1 signaling pathway in PC-12 cells. 

With the multiple protective effects of LCB in anti-oxidative stress-induced ROS production and cell death, the mechanistic action of LCB was further correlated with the induction of autophagy. While MDC can specifically label autophagosomes through ion trapping and specific binding to membrane lipids, the number of autophagic vacuoles on H_2_O_2_-induced PC-12 cells (with or without LCB treatment) revealed by MDC staining further confirmed the autophagic role of LCB in vitro. Again, the AMPK inhibitor, CC, could significantly abolish the protective effect of LCB in rescuing H_2_O_2_-induced apoptosis in PC-12 cells, suggested LCB protected PC-12 cells from H_2_O_2_-induced apoptosis via AMPK. To further validate this conclusion, the level of cleaved–caspase-3, a popular apoptosis protein marker, was restored in LCB-treated H_2_O_2_-induced cells with the presence of CC. While increased autophagy due to ROS production was reported to alleviate apoptosis [37], our findings confirmed the protective autophagic role of LCB in reducing H_2_O_2_-cellular damage in PC-12 cells in a pharmacological approach. While many studies have depicted the molecular role of ATG7 deficiency and impaired autophagy in diseases [38], our results demonstrated that LCB decreased H_2_O_2_-induced apoptosis in ATG7 wild–type cells but not in ATG7-deficient MEFs, suggesting the ATG7-dependent mechanism of LCB. Therefore, with the potential beneficial neuroprotective role LCB in protecting PC-12 cells from H_2_O_2_-induced apoptotic cell death via autophagy, our study has further clarified the traditional therapeutic role of LCB from a pharmacological point of view. However, the autophagic role of LCB in neuronal disease rodent models, and how it can regulate cell survival through the interaction of apoptosis and autophagy, still need to be further investigated.

## 4. Materials and Methods

### 4.1. Reagents and Antibodies

3–(4,5–dimethylthiazol–2–yl)–2,5–diphenyltetrazolium bromide (MTT), paraformaldehyde, bovine serum albumin (BSA), hydrogen peroxide (H_2_O_2_), 2′,7′– dichlorofluorescin diacetate (DCFH–DA), rapamycin, and sodium bicarbonate were obtained from Sigma–Aldrich (Sigma–Aldrich, St. Louis, MO, USA). The cell culture reagents were purchased from Gibco (Grand Island, NY, USA). Licochalcone B was purchased from Herbest Biological Technology Co., Ltd. (Baoji, China). Hoechst 33342, Alexa Fluor 488–labeled goat anti-Rabbit IgG (H + L), the Total Superoxide Dismutase Assay Kit with NBT, Lipid Peroxidation MDA Assay Kit, Calcein/PI Cell Viability/Cytotoxicity Assay Kit, Autophagy Staining Assay Kit with MDC, and LDH Cytotoxicity Assay Kit were purchased from Beyotime Biotechnology Inc. (Shanghai, China). Compound C (CC) was purchased from Topscience Co., Ltd. (Shanghai, China). The Caspase–3 Assay Kit (Colorimetric) was purchased from Abbkine Scientific Co., Ltd. (Wuhan, China). Primary antibodies against apoptosis-related proteins (cleavage–caspase-3, #9664; and caspase-3, #14220) and autophagy-related proteins (LC3, #3868; Beclin–1, #3495; SIRT1, #8469; AMPK, #4150; P–AMPK, #50081; p62, #39749) were obtained from Cell Signaling Technology (Danvers, MA, USA). Anti–GAPDH (sc–47724), β–actin (sc–8432), rabbit (sc–2357), and mouse (sc–2005) IgG–horseradish peroxidase secondary antibodies and an Annexin V Apoptosis Detection Kit (sc–4252 AK) were purchased from Santa Cruz Biotechnology (Santa Cruz, CA, USA).

### 4.2. Cell Culture

PC-12 obtained from the American Type Culture Collection (USA) was cultured in DMEM supplemented with 5% fetal bovine serum, 10% horse serum, 100 μg/mL streptomycin, and 100 U/mL penicillin purchased from Gibco (Grand Island, NY, USA). SH–SY5Y cells were cultured in DMEM supplemented with 10% fetal bovine serum, 100 μg/mL streptomycin, and 100 U/mL penicillin. ATG7−/− and ATG7+/+ cell lines were kindly provided by Masaaki Komatsu (Juntendo University, Tokyo, Japan). All cell lines were placed at a 37 °C incubator supplied with 5% CO_2_.

### 4.3. Establishment of Cellular Oxidative Stress Model 

In brief, PC-12 cells were plated in 6–well (2 × 105 cells/well) plates for overnight cell adhesion. For the detection of cellular oxidative stress, cell death, or mechanisms, 4 different treatment groups were included: (1) Cells pre-treated with different concentrations (from 10 to 40 μM) of LCB for 16 h without H_2_O_2_ treatment (LCB group), (2) cells pre-treated with different concentrations (from 10 to 40 μM) of LCB for 16 h with the addition of 900 μM of H_2_O_2_ for a further 6 h (LCB + H_2_O_2_ treatment group), (3) cells treated with 900 μM of H_2_O_2_ for 6 h (H_2_O_2_ treatment group), and (4) cells treated with solvent or vehicle alone (control treatment group) were set as the experimental conditions. 

### 4.4. Cell Viability Assay

PC-12 cells were plated in a 96–well (5 × 10^3^ cells/well) for overnight cell adhesion. To set up the oxidative stress cellular model, PC-12 cells were treated with 900 μM of H_2_O_2_ for 6 h has described. Cells was then pre-treated with different concentrations of LCB for 16 h prior to the exposure of H_2_O_2_. Then, 10 μL of MTT solution (Sigma, St. Louis, MO, USA) was added into each well for a further incubation of 4 h at 37 °C. To dissolve the formazan, 150 μL of DMSO was added to each well and with its absorbance read by using the microplate spectrophotometer at 490 nm.

### 4.5. Lactate Dehydrogenase Release Assay

Cytotoxicity was measured as the level of lactate dehydrogenase (LDH) by using the LDH assay kit (Beyotime, Nantong, China) [39]. In brief, LDH reduced nicotinamide adenine dinucleotide (NAD) to NADH and formazan, which were quantified as the optical density at 490 nm by using the colorimetric method. 

### 4.6. Caspase–3 Activity Assay

PC-12 cells were treated with 900 μM of H_2_O_2_ for 6 h after pre-treated with different concentrations of LCB for 16 h. Cells was trypsinized, centrifuged at 600× *g* for 5 min at 4 °C, and the supernatant was carefully aspirated. Cells were washed twice with 1 mL of PBS and the supernatant removed. Then 5 × 10^6^ cells were re–suspended in 50 µL cell lysis buffer and mixed with 5 µL Ac–DEVD–pNA. The plate was incubated for 60 min at 37 °C and with the optical density read at 405 nm by using the colorimetric method.

### 4.7. Cellular Autophagy Staining Kit

The induction of autophagy by LCB in PC-12 cells was quantitated by the autophagy staining kit which utilized monodansylcadaverine (MDC) as a fluorescent dye for the detection of autophagic vacuoles. In brief, 1 mL of MDC staining solution was added into each well and incubated with cells for 30 min at a 37 °C incubator and protected from light. The final FITC fluorescent signal was detected by flow cytometry (BD Pharmingen, San Diego, CA, USA).

### 4.8. Hoechst 33342 Staining 

After LCB treatment, PC-12 cells seeded on the cover slides were washed with PBS buffer, fixed with 10% formaldehyde at room temperature for 10 min, and then stained with Hoechst 33342 solution in the dark for 5 min. After mounting, stained nuclei on the slides were observed under the fluorescence microscope (LEICA DM2500, Leica, Wetzlar‎, Germany).

### 4.9. Quantification of Superoxide Dismutase (SOD) and Malondialdehyde (MDA)

PC-12 cells were cultured in a 10 cm dish (5 × 10^5^ cells) and pre-treated with different concentrations of LCB for 16 h. The cells were then exposed to H_2_O_2_ (900 μM) for 6 h. SOD and MDA level in cells were evaluated by using spectrophotometry according to the manufacturer’s assay protocol (Beyotime Institute of Biotechnology, Shanghai, China). The SOD activity was measured at the wavelength of 560 nm, and the level of MDA was detected by using the thiobarbituric acid method with the final absorbance measured at 532 nm [40].

### 4.10. Quantitation of Cellular Apoptosis

Apoptosis was detected by using an FITC Annexin V Apoptosis Detection Kit (BD Biosciences, Franklin Lakes, NJ, USA). After LCB treatment for 16 h, PC-12 cells were washed with PBS and incubated with 100 μL of binding buffer containing annexin V–FITC and propidium iodide in the dark for 30 min at room temperature. The stained samples were analyzed by the FACS Calibur flow cytometer and with the results analyzed by the software Flow Jo 10.

### 4.11. Quantitation of ROS Production

ROS production was determined by using the 2,7–dichlorodihydrofluorescin diacetate (DCFH2–DA) staining assay (Abcam, Cambridge, UK). After pre-treatment of LCB for 16 h, PC-12 cells were incubated with 10 μM DCFH2–DA at 37 °C for 30 min before the induction of H_2_O_2_ (900 μM) for 6 h. The PC-12 cells were re–suspended in PBS and analyzed by flow cytometry for the intensity of the FITC signal. The percentage of fluorescence–positive cells was recorded on a flow cytometer using excitation and emission filters of 488 and 525 nm, respectively.

### 4.12. Quantitative Real–Time Polymerase Chain Reaction (PCR)

After compound treatments, RNA was extracted from PC-12 cells, and the reverse transcription for cDNA was carried out by following the instructions of the cDNA synthesis kit (Transgene, Beijing, China). PCR was performed by using the FastTaq DNA Polymerase Kit (Transgene, Beijing, China). Each PCR reaction was prepared by adding TransScript SuperMix, gDNA Remover, RNase–free water, and 50 ng cDNA templates into a total volume of 25 μL. The real time PCR cycling protocol was performed at 94 °C for 5 min, followed by 40 cycles of 94 °C for 30 s, 60 °C annealing for 1 min, and with a final extension for 10 min at 72 °C by using the Vii7 ABI thermal cycler. 

### 4.13. RT2 Profiler PCR Array

SH–SY5Y cells were treated with or without 20 μM LCB for 24 h. Then, 1 μg of RNA was extracted and reverse transcripted into cDNA. Then, 20 μL cDNA was added per well for the RT^2^ Profiler PCR array analysis on human autophagy genes (Qiagen, Cat. no. PAHS–084Z). The Ct values were uploaded and analyzed by using the software available at the official website (http://www.qiagen.com/geneglobe (accessed on 8 July 2022)) with reference to the Ct values of the panel of housekeeping genes.

### 4.14. Western Blot Analysis

After treatments, cells were lysed in RIPA lysis buffer with the addition of protease and phosphatase inhibitors for protein extraction. The concentration of the total protein extract was determined by using the Bio–Rad DCTM Protein Assay Kit (Bio–Rad, Hercules, CA, USA). Equal amounts (μg) of total protein lysate were loaded into each well of a 10% SDS–PAGE gel. The separated proteins were transferred to a nitrocellulose (NC) membrane. Membranes were blocked with 5% skim milk in TBST for 1 h at room temperature. Corresponding primary antibodies were added for overnight incubation of protein membrane at 4 °C. After the membrane was washed with TBST, secondary antibody was added to incubate with the membrane at room temperature for 1 h. Actin was used as the loading control for normalization of band intensity. The intensity of the protein signal was detected by using the GE Scanner (Belfast, ME, USA).

### 4.15. Gene Enrichment Analysis

The enrichment analysis was performed by STRING (https://string–db.org/) (accessed on 8 July 2022). Gene data collected were further listed (ATG10, ATG4B, ATG4C, ATG5, BID, CTSD, DRAM2, GABARAPL1, HGS, IFNG, INS, MAP1LC3B, NPC1, PIK3CG, RB1, B2M, SIRT1, and AMPK) and input into STRING. Gene correlation mapping was then generated according to enrichment scoring provided by STRING. The results of GO and KEGG pathway were considered for further analysis. Different colors of lines representing different gene interactions and source of data were presented.

### 4.16. Autophagy Assays in C. elegans

Autophagy assays in *C. elegans* were conducted as previously reported [41]. Briefly, the NGM medium of control and experimental groups was added with 100 μL of OP50 *E. coli* bacterial solution, blown dry, and set aside. The synchronized *C. elegans* DA2123 (LGG–1::GFP) and BC12921 (SQST–1/p62::GFP) were placed in an incubator at 20 °C for 16–20 h, centrifuged at 3000 rpm for 2 min, then with most of the supernatant removed until ~1 mL of liquid was left. Then 10 μL of the sample was taken for microscopic observation and counting. According to the calculation of 80 *C. elegans* per NGM dish, the appropriate volume of liquid was added to the NGM dishes with or without 100 μM of LCB or rapamycin (20 μM) and then placed in the incubator at 20 °C after blowing dry on the ultra–clean bench for 48 h. The appropriate number of nematodes was picked and photographed under a fluorescence microscope (100× oil microscope) after anesthesia. Their fluorescence intensity was statistically analyzed.

### 4.17. Statistical Analysis

Data involved in the analysis of variance were obtained from at least 3 independent experiments. All data were expressed as mean and standard deviation (S.D.). Comparisons between the two groups in the experiment were statistically determined by using Student’s *t*–tests. Comparisons between three and more were calculated by one–way analysis of variance (ANOVA) (GraphPad Prism 8.4, San Diego, CA, USA).

## 5. Conclusions

In conclusion, LCB has protective effects against H_2_O_2_-induced PC-12 cell death by reducing the apoptosis rate, LDH release, caspase-3 activity, ROS and MDA level, and protein expression of cleaved caspase-3/caspase-3 and p62. LCB also increased the level of SOD, MDC, LC3II/LCI ratio, Beclin–1, SIRT1, and P–AMPK in both PC-12 cells and *C. elegans* models. Our findings revealed that LCB protected cells from H_2_O_2_-induced apoptosis in an ATG7-dependent manner and via the SIRT1/AMPK signaling pathway, which has provided therapeutic implications for this herbal agent in reducing oxidative damage via neuroprotective autophagic mechanisms in neuronal cells.

## Data Availability

Data is contained within the article and Supplementary Materials.

## Supplementary Materials

The following supporting information can be downloaded at: https://www.mdpi.com/article/10.3390/ph15091052/s1, Figure S1: Establishment of cellular oxidative stress models; Figure S2: DCFH-DA assay for the measurement of ROS level; Figure S3: Western blot for protein expression of caspase-3; Figure S4: Western blot for protein expression of LC3 I/II; Figure S5: Mechanistic study on LCB by Western blot; Figure S6: MDC assay for autophagy detection.
