# Supplementary material for: Licochalcone B, a Natural Autophagic Agent for Alleviating Oxidative Stress-Induced Cell Death in Neuronal Cells and Caenorhabditis elegans Models
Source: Pharmaceuticals (Basel). 2022 Aug 25;15(9):1052. doi: 10.3390/ph15091052 (PMC9502728; doi:10.3390/ph15091052)
Supplement: Supplementary file 1 [file pharmaceuticals-15-01052-s001.zip › pharmaceuticals-1854250-supplementary.pdf]

Supplementary figures for

Original article

Licochalcone B, a Natural Autophagic Agent for Alleviating Oxidative Stress-Induced Cell Death in Neuronal Cells and *Caenorhabditis elegans* Models

Liqun Qu [1,†], Jianhui Wu [1,†], Yong Tang [1,2], Xiaoyun Yun [1], Hang Hong Lo [1], Lu Yu [2], Wenhua Li [3], Anguo Wu [2,*] and Betty Yuen Kwan Law [1,*]

[1] Neher's Biophysics Laboratory for Innovative Drug Discovery, State Key Laboratory of Quality Research in Chinese Medicine, Macau University of Science and Technology, Macau 999078, China
[2] Sichuan Key Medical Laboratory of New Drug Discovery and Druggability Evaluation, School of Pharmacy, Southwest Medical University, Luzhou 646000, China
[3] Hubei Key Laboratory of Cell Homeostasis, College of Life Sciences, Wuhan University,
Wuhan 430072, China
* Correspondence: wuanguo@swmu.edu.cn (A.W.); yklaw@must.edu.mo (B.Y.K.L.)
† These authors contributed equally to this work.

Supplementary Figure S1. Establishment of cellular oxidative stress models

(A) Cell viability of PC-12 cells treated with 0 to 40 μM of LCB for 24 h were measured by MTT assay. (B) Cell viability, (C) LDH level and (D) Caspase-3 activity were measured in H_2O_2 (900 μM) induced PC-12 cells for 6 h after LCB (10, 20 or 40 μM) pre-treatment for 16 h. ###P<0.001, H_2O_2 group vs control; *** P<0.001, H_2O_2 +LCB group vs H_2O_2 group. (E) Cellular morphological changes were evaluated in H_2O_2 (900 μM) induced PC-12 cells for 6 h after pretreatment of LCB for 16 h as indicated. Scale bar =50 μm. (F) Calcein/PI assay was adopted to perform the ICC staining in H_2O_2 (900 μM) induced PC-12 cells for 6 h after pretreatment of LCB for 16 h as indicated. Scale bar =50 μm.

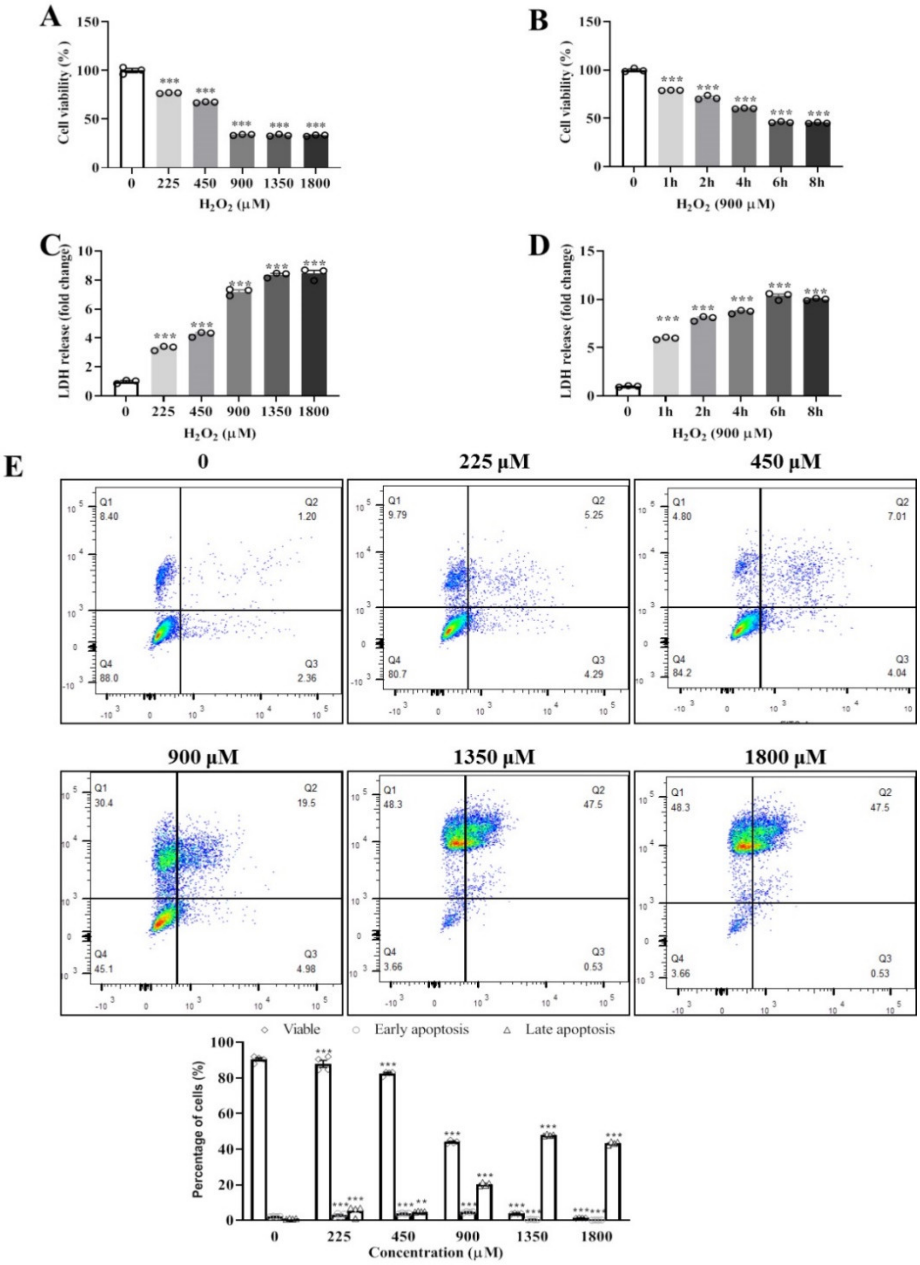

Fig. S1 The establishment of H_2O_2-induced cell death model

Supplementary Figure S2. DCFH-DA assay for the measurement of ROS level

PC-12 cells were pretreated with 25 μM of LCB for 16 h before the exposure of 900 μM H_2O_2 for 1 h. Intracellular ROS production was determined by using the 10 μM DCFH-DA assay. Scale bar= 50 μm.

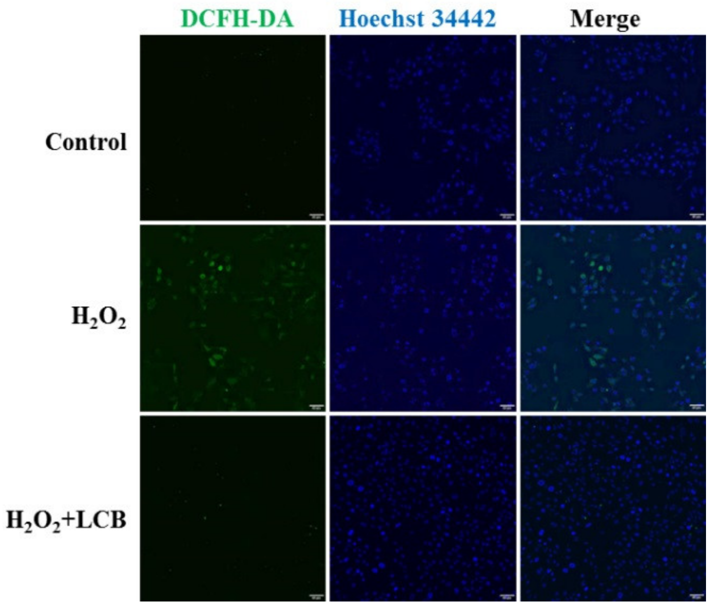

Fig. S2 Effect of LCB on H_2O_2-induced oxidative stress in PC-12 cells

Supplementary Figure S3. Western blot for protein expression of caspase 3

PC-12 cells were pretreated with LCB (10 to 40 μM) for 16 h before subjected to 900 μM H_2O_2 for 6h. Western blot analysis was used to detect the level of cleaved or total caspase 3 protein expression, *###P<0.001, H_2O_2 group vs control; *** P<0.001, H_2O_2 +LCB group vs H_2O_2 group.*

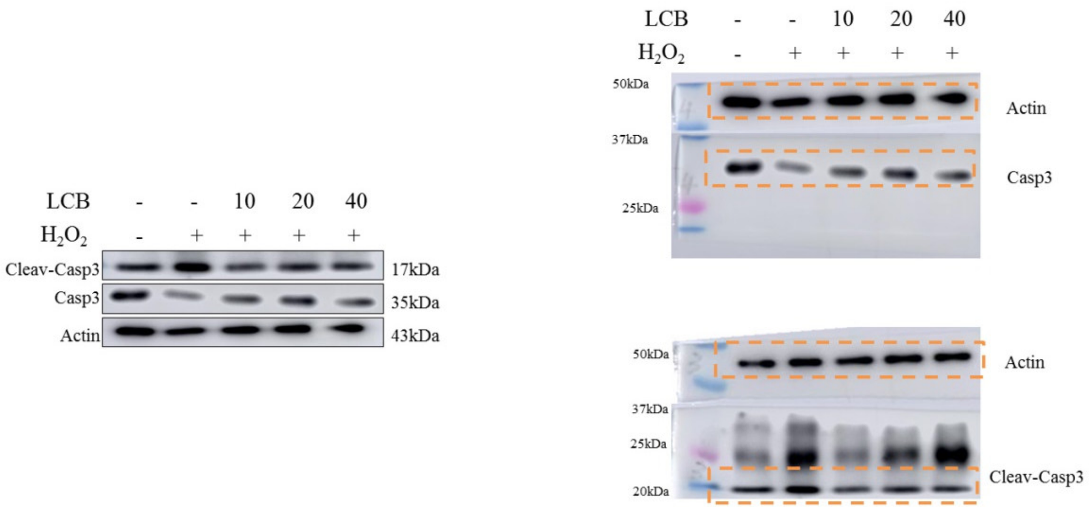

Fig. S3 LCB protected PC-12 cells from apoptosis induced by H_2O_2

Supplementary Figure S4. Western blot for protein expression of LC3 I/II

GFP-LC3-U87 cells were exposed to various concentrations of LCB (10 to 40 μM) for 24 h as indicated. Western blot for the expression of LC3-II/I after treatment of LCB or rapamycin for 24 h. * P<0.01, *** P<0.001.

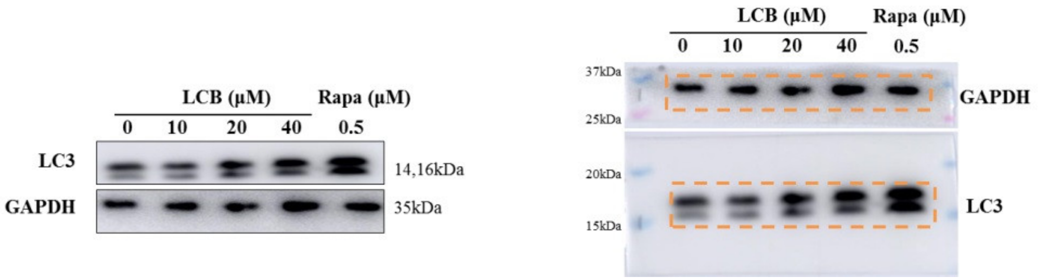

Fig. S4 LCB induced autophagy in GFP-LC3-U87 cells

Supplementary Figure S5. Mechanistic study on LCB by Western blot.

(A) PC-12 cells were treated with LCB under the indicated concentrations for 24 h. After treatment, cell lysates were harvested for the analysis of SIRT1, P-AMPK, Beclin1, and p62 by Western blot. (B) PC-12 cells were treated with LCB with or without the presence of CC under the indicated concentrations for 24 h. After treatment, cell lysates were harvested for the analysis of SIRT1, P-AMPK, and p62 and LC3 by Western blot.

(A)

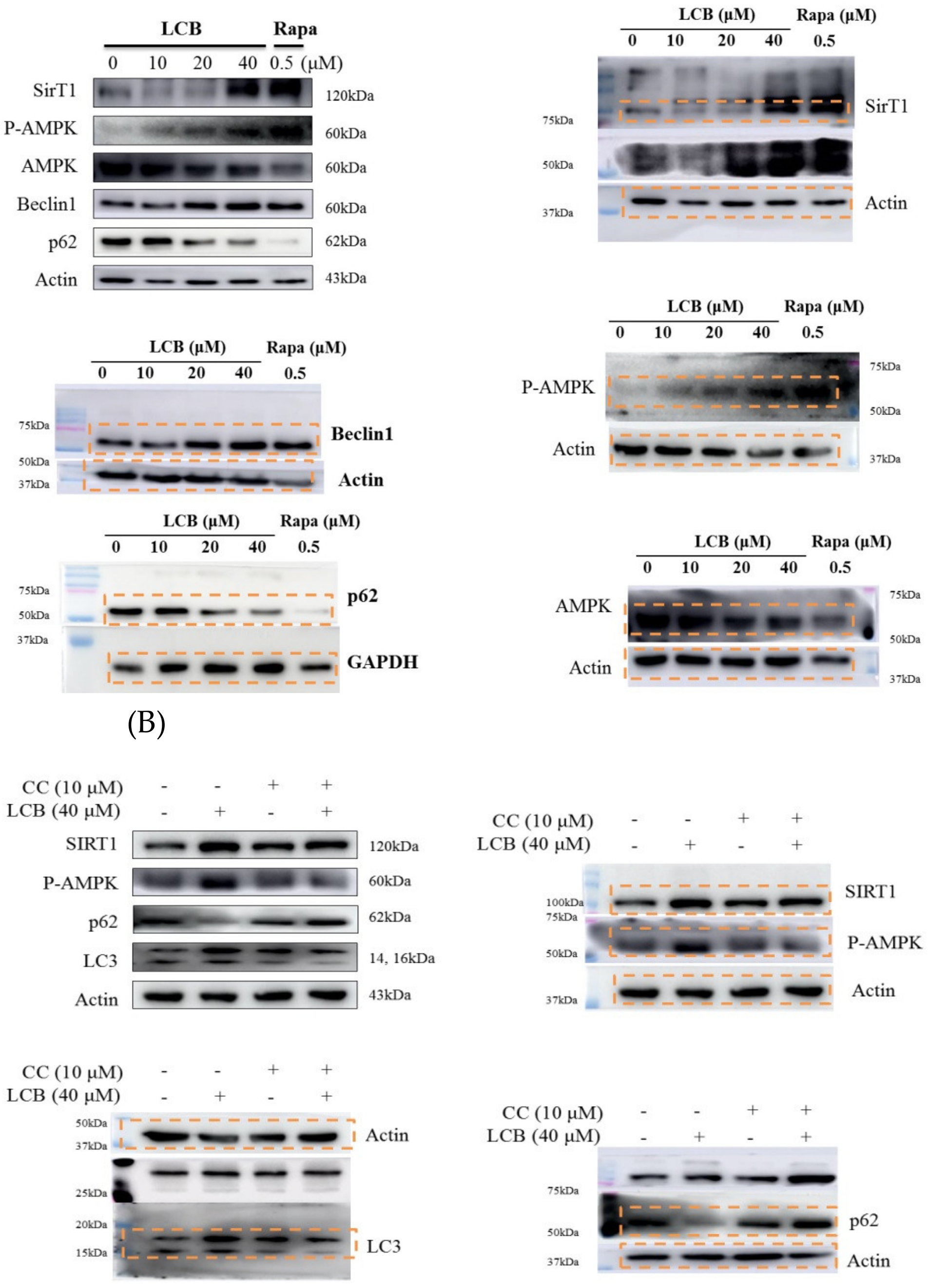

Fig.S5 LCB activated autophagy via the AMPK/SIRT1 signaling pathway in PC-12 cells

Supplementary Figure S6. MDC assay for autophagy detection

PC-12 cells were pretreated with 10 to 40 μM of LCB for 16 h before the exposure to 900 μM H_2O_2 for 6 h. MDC staining buffer was added and incubated with cells for 30 min at 37°C incubator protected from light. *** *p<0.001.*

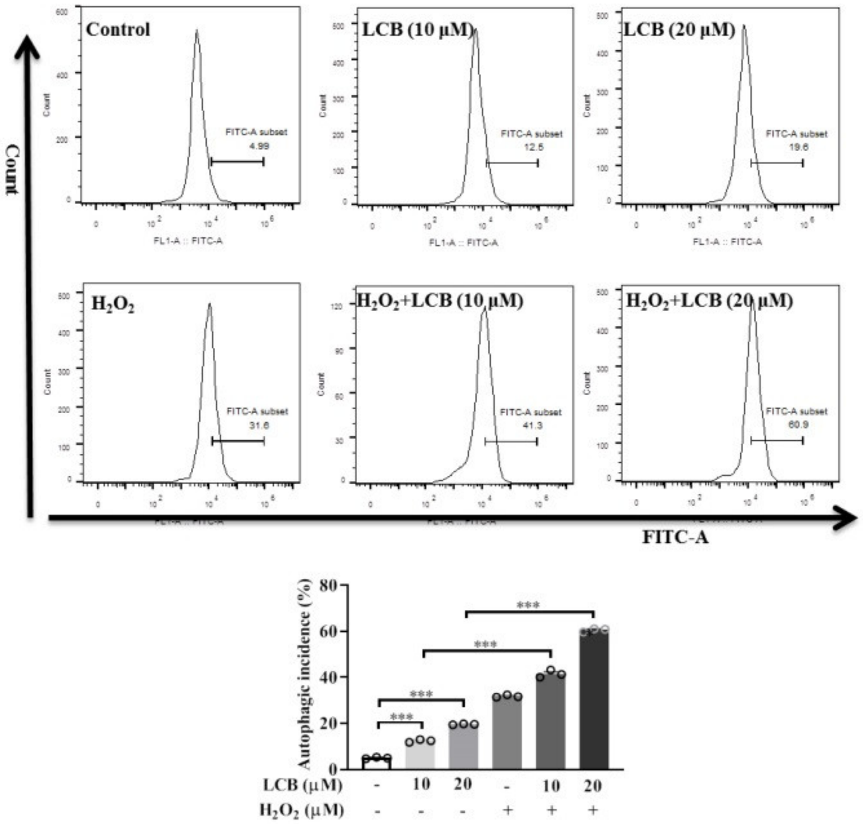

Fig.S6 The induction of autophagy in LCB treated H_2O_2-induced PC-12 cells by MDC staining and flow analysis
